# Population connectivity of pelagic megafauna in the Cuba-Mexico-United States triangle

**DOI:** 10.1038/s41598-018-38144-8

**Published:** 2019-02-07

**Authors:** Jay R. Rooker, Michael A. Dance, R. J. David Wells, Matthew J. Ajemian, Barbara A. Block, Michael R. Castleton, J. Marcus Drymon, Brett J. Falterman, James S. Franks, Neil Hammerschlag, Jill M. Hendon, Eric R. Hoffmayer, Richard T. Kraus, Jennifer A. McKinney, David H. Secor, Gregory W. Stunz, John F. Walter

**Affiliations:** 1 Department of Marine Biology, Texas A&M University, 1001 Texas Clipper Road, Galveston, Texas 77554 USA; 2 Department of Wildlife and Fisheries Sciences, Texas A&M University, College Station, Texas 77843 USA; 3 Department of Oceanography and Coastal Sciences, Louisiana State University, 2255 Energy, Coast and Environment Building, Baton Rouge, Louisiana 70803 USA; 4 Harbor Branch Oceanographic Institute, Florida Atlantic University, 5600 US 1 North, Fort. Pierce, Florida 34946 USA; 5 Hopkins Marine Station, Stanford University, 120 Ocean View Blvd., Pacific Grove, California, 93950 USA; 6 Mississippi State University, Coastal Research and Extension Center, 1815 Popps Ferry Road, Biloxi, Mississippi 39532 USA; 7 Louisiana Department of Wildlife and Fisheries, 2021 Lakeshore Dr., Suite 220, New Orleans, Louisiana 70122 USA; 8 Gulf Coast Research Laboratory, University of Southern Mississippi, 703 East Beach Drive Ocean Springs, Mississippi, 39564 USA; 9 Rosenstiel School of Marine & Atmospheric Science, University of Miami, 4600 Rickenbacker Causeway, Miami, Florida 33149 USA; 10 NOAA Fisheries, Southeast Fisheries Science Center, Mississippi Laboratories, P.O. Drawer 1207, Pascagoula, Mississippi 39568 USA; 11 Lake Erie Biological Station, USGS, 6100 Columbus Avenue, Sandusky, Ohio, 44870 USA; 12 Chesapeake Biological Laboratory, University of Maryland Center for Environmental Science, P.O. Box 38, Solomons, Maryland, 20688 USA; 13 Harte Research Institute for Gulf of Mexico Studies, Texas A&M University-Corpus Christi, 6300 Ocean Drive, Unit 5869, Corpus Christi, Texas 78412 USA; 14 NOAA Fisheries, Southeast Fisheries Science Center, 75 Virginia Beach Drive, Miami, Florida 33149 USA

## Abstract

The timing and extent of international crossings by billfishes, tunas, and sharks in the Cuba-Mexico-United States (U.S.) triangle was investigated using electronic tagging data from eight species that resulted in >22,000 tracking days. Transnational movements of these highly mobile marine predators were pronounced with varying levels of bi- or tri-national population connectivity displayed by each species. Billfishes and tunas moved throughout the Gulf of Mexico and all species investigated (blue marlin, white marlin, Atlantic bluefin tuna, yellowfin tuna) frequently crossed international boundaries and entered the territorial waters of Cuba and/or Mexico. Certain sharks (tiger shark, scalloped hammerhead) displayed prolonged periods of residency in U.S. waters with more limited displacements, while whale sharks and to a lesser degree shortfin mako moved through multiple jurisdictions. The spatial extent of associated movements was generally associated with their differential use of coastal and open ocean pelagic ecosystems. Species with the majority of daily positions in oceanic waters off the continental shelf showed the greatest tendency for transnational movements and typically traveled farther from initial tagging locations. Several species converged on a common seasonal movement pattern between territorial waters of the U.S. (summer) and Mexico (winter).

## Introduction

Large pelagic fishes are common apex predators in coastal and open ocean ecosystems^1,2^ and play important roles in structuring marine communities through top-down control^3,4^. Conservation and rebuilding efforts for key constituents of the pelagic fish assemblage (e.g., billfishes, tunas, and sharks) requires species-specific information on movements (i.e., spatial displacements) necessary for individuals to complete their life cycles^5,6^. This is due to the fact that overexploitation and incidental bycatch are arguably the most critical barriers to conserving and rebuilding billfish, tuna, and shark populations^7,8^, and these threats vary both spatially and temporally^9^. As a result, an improved understanding of the spatial dynamics and movement pathways/phases of these predators is needed to support both sustainable fisheries and the conservation of pelagic ecosystems^10,11^. Because billfishes, tunas, and sharks routinely traverse international borders and high sea regions, migrations respective to boundaries are often key in implementing effective management strategies^12^.

Tri-national initiatives among Cuba, Mexico, and the United States (U.S.) are currently being developed to advance the conservation of marine ecosystems and pelagic fisheries in the Gulf of Mexico (GoM)^13,14^. Billfishes, tunas, and sharks are common components of the pelagic ecosystem in the GoM^15–17^, and the territorial waters of the three countries serve as critical spawning, nursery, and/or foraging habitat for multiple species within each taxonomic group^18–20^. Conservation measures for these pelagic predators vary spatially across the GoM^7,10^, with each country displaying different levels of cooperation in fishery organizations responsible for their management. As an example, Mexico and the U.S. are contracting parties of the International Commission for the Conservation of Atlantic Tunas (ICCAT), while Cuba’s participation in the Commission ended in 1991. In response, international crossings both within and outside the GoM will expose individuals to different levels of fishing pressure (e.g. pelagic longline effort), which in turn can have profound impacts on a species’ population dynamics^16^.

**Figure 1:**
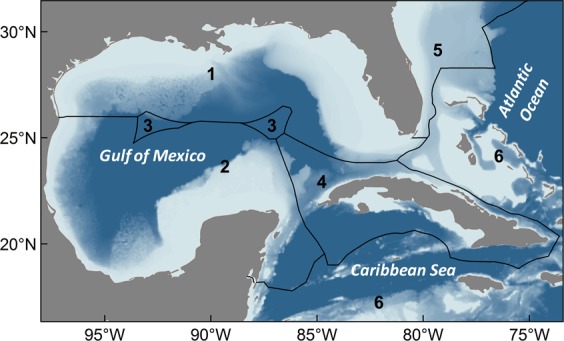
Map showing the 6 designated regions used to assess the population connectivity of pelagic predators: (1) U.S. GoM (includes Florida Keys), (2) Mexico (all territorial waters in the GoM plus waters near the Yucatan Channel), (3) High Seas areas in the GoM, (4) Cuba (all territorial waters), (5) U.S. waters in the Atlantic Ocean (east coast), and (6) all remaining areas in the Atlantic Ocean outside regions 1–5.

Here, we characterize the spatial and temporal (seasonal) distribution of selected pelagic fishes in the GoM to better understand the significance of population connectivity and use of the territorial waters within the Cuba-Mexico-U.S. triangle. Our investigation is based on electronic tagging research conducted in U.S. waters of the GoM (hereafter U.S. GoM) (Fig. 1), and includes tag deployments on three general categories of pelagic predators: (1) billfishes, (2) tunas, and (3) sharks. The first two taxonomic categories include four marine teleosts (bony fishes) of high commercial and ecological value in pelagic ecosystems: blue marlin (*Makaira nigricans*), white marlin (*Kajikia albida*), Atlantic bluefin tuna (*Thunnus thynnus*), and yellowfin tuna (*T*. *albacares*). Coastal and open ocean migratory sharks investigated here are equally important from an ecological point of view and selected species range from the filter feeding whale shark (*Rhincodon typus*) to upper-level predators: scalloped hammerhead (*Sphyrna lewini*), shortfin mako (*Isurus oxyrinchus*), and tiger shark (*Galeocerdo cuvier*). Several species included in our assessment are currently red listed as “endangered” (Atlantic bluefin tuna, whale shark, scalloped hammerhead) by the International Union for the Conservation of Nature (IUCN), with three other species listed as “vulnerable” (white marlin, blue marlin, shortfin mako^21^), further emphasizing the significance of this study. The primary goal of this investigation was to quantify the timing and prevalence of international crossings displayed by each species as well as identify areas of high exchange or crossing hotspots.

## Results

Population connectivity of billfishes (n = 65), tunas (n = 98), and pelagic sharks (n = 133) was based on electronic tagging data collected from 2003 to 2018 (Table 1). The total number of positional tracking days (daily position estimates) used for all eight species investigated was substantial (22,289 daily positions), and reasonably extensive for each taxonomic group: billfishes (5,332 daily positions), tunas (10,105 daily positions), and sharks (6,852 daily positions) (Table 1). Overall, the spatial distribution of these taxa within the GoM and the incidence of international crossings varied considerably among the eight species investigated but demonstrated that territorial waters of all three countries were visited, often regularly, by most of these pelagic predators.

**Table 1:** Summary of electronic tags deployed on the eight pelagic predators investigated.

Species	Period	Tagging location	N	Tag type	Number of daily positions	Mean ‘days at liberty’ (±SE)
**Billfishes**						
Blue marlin	2003–2015	NC, NW GoM	59	PAT^MT,WC^	4432	119 ± 15
White marlin	2009–2015	NC, NW GoM	6	PAT^MT^	900	187 ± 63
**Tunas**						
Atlantic bluefin tuna	2001–2012	NC GoM, Canada	44	PAT^WC^	6710	153 ± 14
Yellowfin tuna	2008–2016	NC GoM	54	PAT^MT,WC^, AIT^LT^	3395	73 ± 11
**Sharks**						
Whale shark	2009–2015	NC GoM	42	PAT, SPOT	2764	104 ± 16
Tiger shark	2011–2018	NC, NE, NW GoM	54	PAT^WC^, SPOT	1714	94 ± 16
Scalloped hammerhead	2012–2016	NC, NE, NW GoM	33	SPOT	1690	146 ± 24
Shortfin mako	2016–2018	NW GoM	4	SPOT	684	272 ± 154

Tagging period, location (NW = northwestern, NC = northcentral, NE = northeastern) in the Gulf of Mexico (GoM), number of tagged individuals (N), and tag type denoted. Number of estimated daily positions available and mean ‘days at liberty’ (±1 standard error) provided for each species.

Superscript denotes manufacturer for pop-up archival tag (PAT), smart position tag (SPOT) and archival implant tag (AIT): ^WC^Wildlife Computers (MK-10 PAT, Mini-PAT), ^MT^Microwave Telemetry (X Tag), ^LT^Lotek Wireless (LAT Series).

### Billfishes

**Figure 2:**
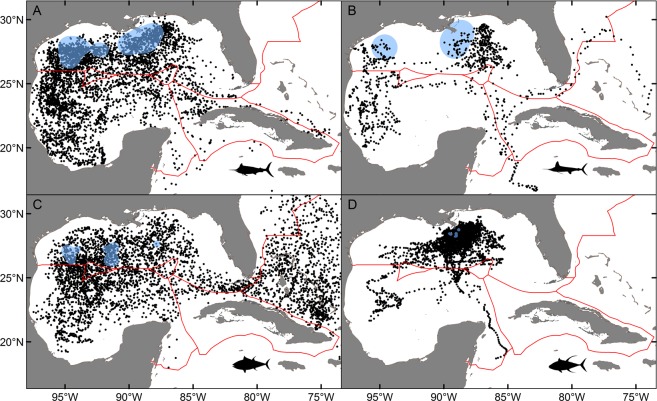
Daily position estimates for billfishes and tunas tagged: (**A**) blue marlin, (**B**) white marlin, (**C**) Atlantic bluefin tuna, (**D**) yellowfin tuna. Primary tagging areas for each species are shown as 95% kernel utilization distributions (blue shading). Atlantic bluefin tuna also included tagging conducting in Canada. Red lines indicate territorial boundaries.

**Figure 3:**
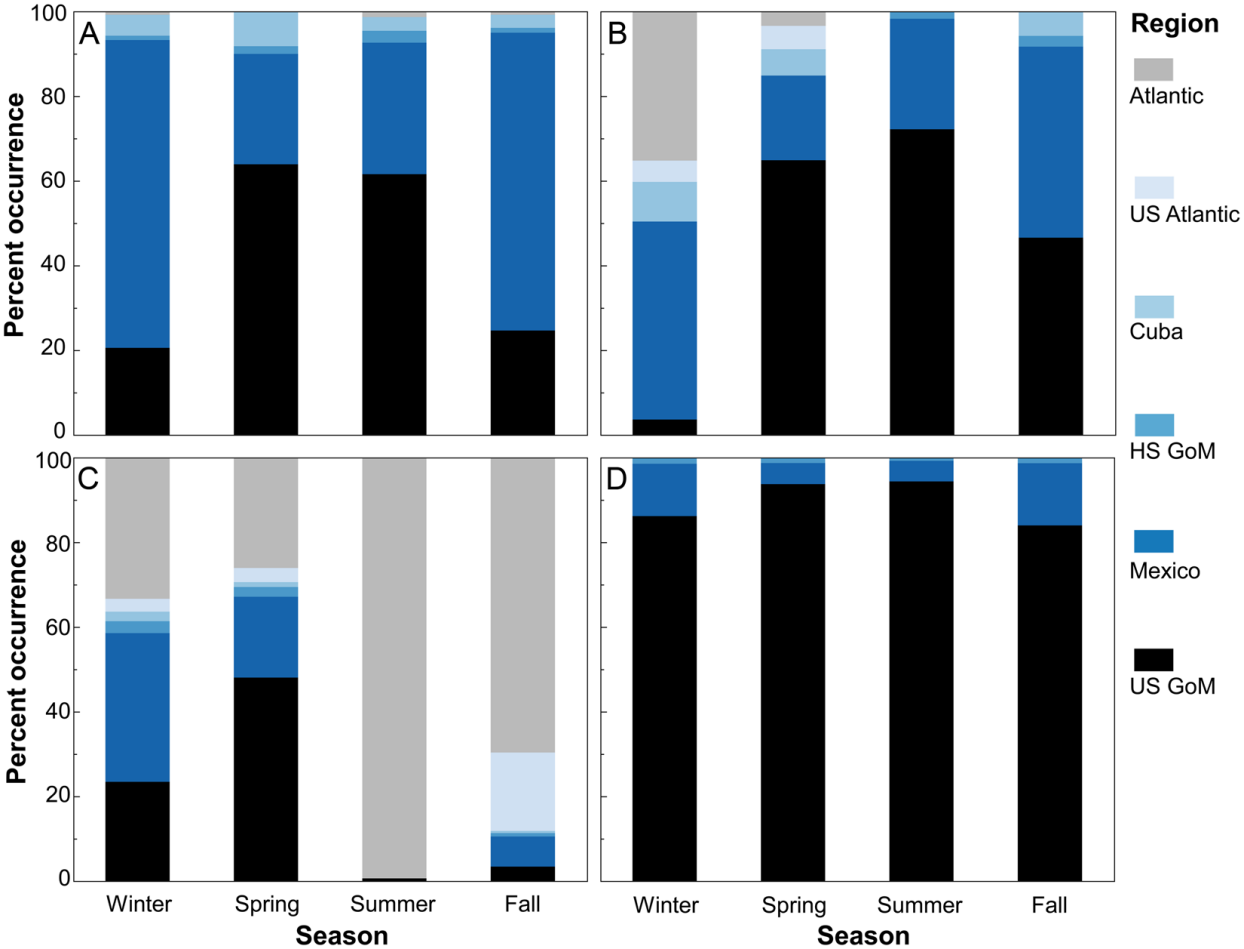
Percent occurrence by season in the 6 designated regions for billfishes and tunas: (**A**) blue marlin, (**B**) white marlin, (**C**) Atlantic bluefin tuna, (**D**) yellowfin tuna. Estimates for winter, spring, summer, and fall are based on all daily position estimates within that season for each species.

Tagging data demonstrated strong bi-national connectivity for blue marlin between Mexico and the U.S. with nearly half of the overall daily positions in the territorial waters of Mexico, primarily in the southern GoM (Bay of Campeche) or off the Yucatan Peninsula. Blue marlin also crossed into the territorial waters of Cuba, often passing through the Straits of Florida and moving into areas off eastern Cuba near Haiti (Fig. 2A) or farther north into the Bahamas. Movements of blue marlin were essentially restricted to Cuba, Mexico, and the U.S. GoM; however, daily positions were also present farther east into the Caribbean Sea after egress through the Straits of Florida or Yucatan Channel. Although this species is capable of long-distance displacement (>1000 km), movement was largely restricted to the GoM and the passageways connecting this basin to the Atlantic Ocean. In addition, no individuals were detected north of Miami, Florida in U.S. waters or farther east in the Atlantic Ocean. International crossings varied seasonally with the highest occurrence of blue marlin in the U.S. GoM (61.6%) during summer (July-September), followed by individuals moving into the territorial waters of Mexico in fall (October-December) and winter (January-March) (Fig. 3A). In fact, over 70% of the daily positions for blue marlin in fall (70.3%) and winter (72.7%) were from positions in the southern GoM (Mexico). Movement back into the U.S. GoM occurred during spring (April-June) with the majority of daily positions (63.9%) once again in the northern part of the basin.

Data for white marlin were more limited due to a smaller sample size, but results further underscore the importance of transnational movements. Daily positions for white marlin were observed in the territorial waters of both Cuba and Mexico, with movements into Mexico being reasonably common (Fig. 2B). Given that nearly all tagging was conducted in the U.S. GoM from May to September, it was not unexpected that daily position estimates were highest in this region during summer (75.5%). Nevertheless, seasonal shifts were clearly evident again, and the largest percentage of daily positions in fall (45.1%) and winter (46.8%) seasons occurred in the territorial waters of Mexico (Fig. 3B), which may signify a movement pattern similar to blue marlin (i.e., overwintering in the southern GoM). Daily positions observed in waters off Cuba also peaked in winter (9.4%) with positions also detected north of Cuba in the Bahamas, indicating that this area may also represent overwintering habitat.

### Tunas

Atlantic bluefin tuna tagged in the U.S. GoM or farther north (Canada) but returning to this region to spawn were common in the territorial waters of both Mexico and the U.S. GoM (Fig. 2C). High occurrence of this species was observed throughout the northwestern GoM, particularly in outer shelf and slope waters both north and south of the Mexico-U.S. territorial boundary at ~26°N latitude. Daily positions were relatively rare in the territorial waters of Cuba but more common in areas to the north in the Bahamas. Because Atlantic bluefin tuna display directed migrations between spawning areas (GoM) and foraging areas (North Atlantic Ocean), our assessment of temporal variability in daily positions within Cuba, Mexico, and the U.S. GoM was primarily limited to periods when they occupy this basin, January to June, which corresponds to winter (January-March) and spring (April-June) seasons in this region. During winter and spring, percentages of daily positions in the territorial waters of Mexico and the U.S. GoM combined were 57.5% and 67.1%, respectively. We observed a higher overall percentage of daily positions in Mexico during winter (35.1%) and this trend was reversed in spring with a higher percentage occurring in the U.S. GoM (48.0%), probably representing a northward shift by Atlantic bluefin tuna as individuals get ready to spawn (Fig. 3C). Spawning adults exit the basin as the water temperature in the GoM begin to increase, and nearly all of the daily positions in summer (July-September) were outside the GoM in the Atlantic Ocean (97.8%) with only 2.2% of the daily positions in the U.S. GoM. This general trend continued into fall with a small percentage of daily positions still present in either Mexico (7.1%) or the U.S. GoM (3.4%), although most of these daily positions were from a single month (December) and represent early entry into the spawning area by a few individuals.

International crossings by yellowfin tuna in the GoM were less evident relative to the other teleosts investigated (Fig. 2D). Even though tagging data for yellowfin tuna was based on a fairly large sample size (>50 individuals tracked) and included several fish with relatively long deployment periods (9 tags 6–12 + month tracking periods), 89.5% of the daily positions for yellowfin tuna were in the U.S. GoM; all remaining positions were either in Mexico (9.3%) or the two high seas regions in the GoM (1.2%). Thus, transnational movements by yellowfin tuna were limited entirely to Mexico, with no individuals entering the territorial waters of Cuba or areas in the western Atlantic Ocean. The spatial distribution of daily positions intimated the potential for longer distance migration through the Yucatan Channel (i.e., outside GoM), but these positions were based on a single individual. Similar to blue marlin and white marlin, the percentage of daily positions for yellowfin tuna in Mexico increased during fall (14.7%) and winter (12.4%) relative to spring (5.0%) and summer (0.0%) (Fig. 3D), again conveying that a fraction of the GoM population may overwinter in Mexico. Nevertheless, seasonal shifts were less pronounced for yellowfin tuna than blue marlin, white marlin, or Atlantic bluefin tuna, and retention (i.e. lower spatial displacement) within the northern GoM appears to be remarkably high for individuals tagged in this region.

### Sharks

**Figure 4:**
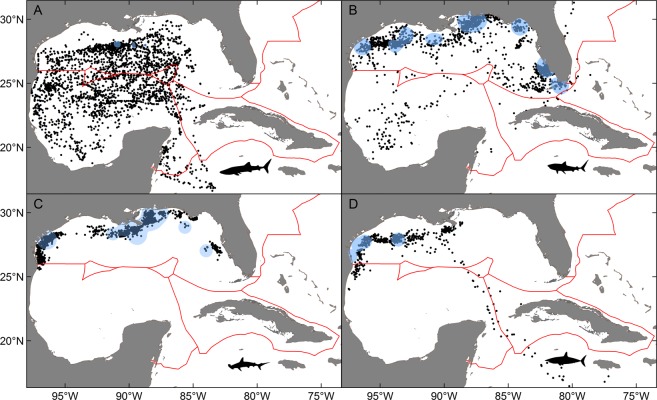
Daily position estimates for sharks tagged: (**A**) whale shark, (**B**) tiger shark, (**C**) scalloped hammerhead, (**D**) shortfin mako. Primary tagging areas for each species are shown as 95% kernel utilization distributions (blue shading). Red lines indicate territorial boundaries.

**Figure 5:**
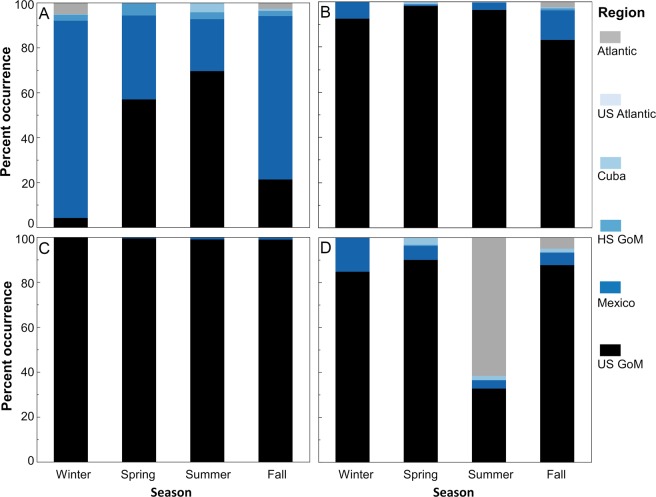
Percent occurrence by season in the 6 designated regions for sharks: (**A**) whale shark, (**B**) tiger shark, (**C**) scalloped hammerhead, (**D**) shortfin mako. Estimates for winter, spring, summer, and fall are based on all daily position estimates within that season for each species.

Daily positions for whale sharks were common in the territorial waters of all three countries, and the majority of daily positions were present on the outer continental shelf/slope or in oceanic waters. Transnational movements were primarily between Mexico and the U.S. GoM, with almost 50% of all daily positions occurring in Mexico even though all tagging was conducted to the north in the U.S. GoM. Most of the remaining daily positions (44.3%) for whale sharks were located in the U.S. GoM, which is further evidence of strong bi-national connectivity between the two countries. Daily positions of whale sharks were relatively widespread throughout the entire basin, including areas within or proximal to the Yucatan Channel in the territorial waters of all three countries (Fig. 4A). International crossings into Cuba were evident near the Yucatan Channel, but daily positions in Cuba only accounted for 2.1% of the total percent occurrence (Fig. 5A). Nearly 2% of the daily positions were outside the territorial waters of all three countries and, similar to billfishes and tunas, long distance movements (>1000 km) occurred for this species. The spatial distribution of whale sharks in the Cuba-Mexico-U.S. triangle varied seasonally with daily positions of whale sharks in the U.S. GoM  peaking in spring (57.0%) and summer (69.6%) (Fig. 5A). Summer occupancy of the northern GoM by whale sharks was followed by shifts into Mexico during fall (77.3%) and winter (87.8%), which again is suggestive of overwintering in more southerly latitudes. In addition, daily positions were also detected in areas south of the Yucatan Channel outside the territorial waters of all three countries in areas off Belize.

The degree of exchange among Cuba, Mexico, and the U.S. for the three other shark species (scalloped hammerhead, shortfin mako, tiger shark) was negligible to modest, although transnational movements were detected for all three species (Fig. 4B–D). Even though the number of tagged individuals and amount of daily positions (685 days) were limited for the shortfin mako relative to both scalloped hammerheads and tiger sharks, this species showed the greatest capacity for moving into waters outside the U.S. GoM (Fig. 5B–D). In fact, nearly 20% of the overall daily positions were from other regions: Cuba (1.8%), Mexico (8.2%), and Atlantic Ocean outside U.S. waters (10.9%). Seasonal shifts were present for shortfin makos with daily positions in Mexico highest during winter (15.3%), which was followed by a conspicuous shift to areas in the Atlantic Ocean during summer (61.7%). However, a large fraction of the daily positions outside the GoM were from a single individual moving through the Yucatan Channel and into the Caribbean Sea as far east as Jamaica. Movements of scalloped hammerheads and tiger sharks were more limited even though the total number of daily positions and deployment durations for both species were comparable or higher than other species included in the assessment (Table 1). Over 99% of all daily positions for scalloped hammerheads were in the U.S. GoM. No salient seasonal patterns were detected with daily positions occurring in the territorial waters of Mexico being insignificant during all seasons (~1% or less). Although a large percentage of the overall daily positions for tiger sharks also occurred in the U.S. GoM (91.7%), a meaningful percentage of daily positions were present in the territorial waters of Mexico during fall and winter seasons (13.1% and 7.6%, respectively). Both scalloped hammerheads and tiger sharks showed very limited or no movement into waters off Cuba or areas outside the GoM.

### Multispecies border crossing locations

**Figure 6:**
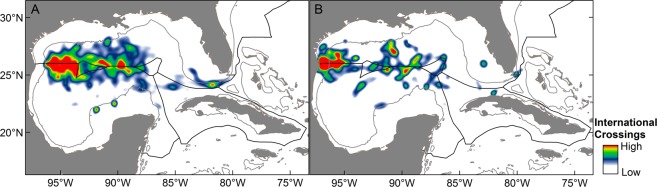
Movement-based kernel density estimates showing hotspots of exchange (i.e., international crossing areas) within the Cuba-Mexico-U.S. triangle for two multispecies groups: (**A**) billfishes and tunas, (**B**) sharks. Utilization distributions based on daily positions estimates 10 days before and 10 days after every international crossing by individuals within each respective multispecies group.

Distinct areas of exchange were identified for our multispecies groups within the Cuba-Mexico-U.S. triangle using movement-based kernel density estimates (MKDE) based on daily position estimates 10 days before and 10 days after each international crossing. Unexpectedly, two relatively large geographic areas of exchange were present for both multispecies groups: (1) western GoM along Mexico-U.S. border and (2) central GoM along 26°N latitude and between both high seas regions (Fig. 6). The largest crossing hotspot for billfishes and tunas occurred in the western GoM and extended from the outer continental shelf to slope waters well beyond the 200-m isobath from approximately 94° to 96°W longitude. A second crossing location for billfishes and tunas was again present along 26°N latitude but farther east from 88° to 90°W longitude. Less conspicuous areas of exchange with Cuba were present in the Straits of Florida. Similar to billfishes and tunas, a key crossing location for the shark multispecies group was identified in the western GoM; however, movement-based distributions of sharks for this region occurred primarily on the continental shelf and closer to shore (inside 200-m isobath), and the frequency of open ocean crossings (off the continental shelf, >200 m depth) was significantly lower for the shark group than observed for billfishes and tunas (*X*^2^ = 27.27, df = 1, p < 0.001) (Fig. 6B). Areas of high exchange along the territorial boundary separating Cuba from both Mexico and the U.S. (~87°W) were also present for sharks but this was largely a function of whale shark movements, which strongly influenced MKDEs for this multispecies group. In contrast to the billfishes and tunas, crossings in areas east of 85°W along Cuba-U.S. boundary including the Straits of Florida were limited for the shark multispecies group.

### International crossings as a function of days at liberty

**Figure 7:**
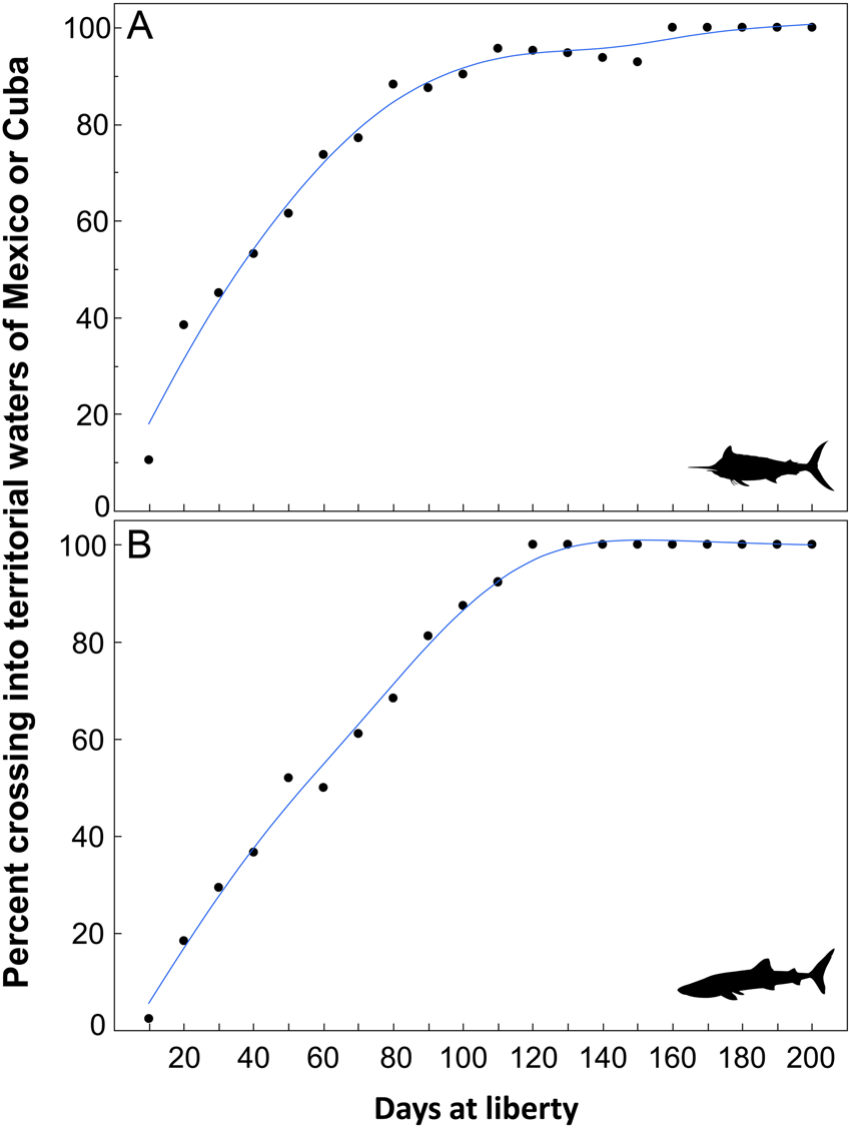
Relationship between days at liberty (i.e., tag duration) and the occurrence of an international crossing from the U.S. GoM into territorial waters of Cuba or Mexico for a representative teleost and shark: (**A**) blue marlin and (**B**) whale shark. Locally weight scatterplot smoothing (LOWESS) was used as the non-parametric regression method to describe the non-linear relationship for each species.

The influence of tag duration on the probability of moving out of the U.S. GoM and into the territorial waters of either Cuba or Mexico was investigated for a representative teleost (blue marlin) and shark (whale shark). The two species were ideal for examining the relationship between days at liberty and international crossings because neither showed residency to the northern GoM, and daily position data were substantial for both species. The prevalence of international crossings by blue marlin and whale sharks into Cuba or Mexico was strongly affected by days at liberty. The percentage of blue marlin visiting either Cuba or Mexico increased rapidly during the first 100 days, ranging from about 10% at 10 days at liberty to over 90% at 100 days at liberty (Fig. 7A). Similarly, international crossings by whale sharks increased rapidly ranging from <5% at 10 days at liberty to nearly 90% at 100 days at liberty (Fig. 7B). After approximately 120 and 160 days at liberty for whale sharks and blue marlin, respectively, 100% of tagged individuals with deployment durations extending this long had entered the territorial waters of Cuba and/or Mexico, suggesting that tracking periods of approximately 150 days or greater appear suitable for assessing international exchange of these and potentially other pelagic fishes in the GoM.

In response, we quantified bi- (occurrence in Cuba or Mexico) and tri-national (occurrence in both Cuba and Mexico) population connectivity for all individuals tagged in the U.S. GoM with tag deployments of 150 days or longer (Table 2). For seven of eight species investigated, at least half of the individuals with days at liberty of 150 days or more showed strong bi-national connectivity, with the one exception being scalloped hammerheads. In addition, 50% or more of the long-term tag deployments (>150 days) for white marlin, Atlantic bluefin tuna, and shortfin mako showed tri-national connectivity, with individuals occupying the territorial waters of Cuba, Mexico, and the U.S. during the deployment period (Table 2). The mean number of regions visited (Fig. 1) for individuals with tracking duration of 150 days or more was also the highest for Atlantic bluefin tuna (5.4) and white marlin (4.7), which was due to many individuals present in the territorial waters of all three countries within the GoM, the high seas region of the GoM, and both regions outside the GoM in the Atlantic Ocean.

**Table 2:** Summary information on population connectivity for individuals with long-term tag deployments (‘days at liberty’ >150 days).

Species	N	Percent (%)		Regions Visited^†^		
		Bi- Nat.	Tri-Nat.	Min	Max	Mean
**Billfishes**						
Blue marlin	14	93	36	1	4	2.9
White marlin	3	100	67	2	6	4.7
**Tunas**						
Atlantic bluefin tuna	26	100	77	4	6	5.4
Yellowfin tuna	8	50	0	1	3	1.9
**Sharks**						
Whale shark	11	100	27	2	4	2.9
Tiger shark	12	33	17	1	6	2.0
Scalloped hammerhead	9	22	0	1	2	1.2
Shortfin mako	2	50	50	1	5	3.0

Percent of individuals displaying bi- and tri-national population connectivity (daily positions in the U.S. plus Cuba or Mexico versus daily positions in all three countries) is provided along with minimum, maximum, and mean number of regions visited by individuals within each species.

^†^6 possible regions: U.S. GoM, Mexico, High Seas areas in the GoM, Cuba, U.S. waters in the Atlantic Ocean outside GoM, and all remaining areas of the Atlantic Ocean.

## Discussion

International crossings by pelagic predators tagged in the U.S. GoM occurred for nearly all of the species investigated with varying levels of bi- or tri-national population connectivity displayed by billfishes, tunas, and sharks. In general, teleosts included in the assessment (billfishes and tunas) commonly moved throughout the basin, with individuals regularly crossing management boundaries (*sensu* ‘crossing the line’^22^) and entering the territorial waters of Cuba or Mexico. Although whale sharks commonly used the territorial waters of Cuba, Mexico and the U.S., the other sharks investigated generally displayed more limited movements with two species (scalloped hammerhead and tiger sharks) exhibiting prolonged periods of residency to areas in the northern GoM. Migration patterns and transnational movements of pelagic fishes at the basin scale are commonly linked to both intrinsic and external factors^1,5^, and observed spatial shifts in distributions for billfishes, tunas, and sharks in the GoM appear to be related to directed movements between foraging and spawning areas as well as exploratory and/or physiologically motivated movements potentially associated with oceanographic conditions^23,24^. Because spawning or related reproductive activities (e.g., mating, parturition) for the majority of species investigated occurs in the GoM^15,25,26^, movements out of the U.S. GoM and/or systematic returns to this area after journeys to Cuba, Mexico and areas outside the basin (i.e., natal homing and/or spawning site fidelity^16,27^) are likely determined by the interplay of intrinsic factors and environmental conditions experienced by individuals.

The prevalence of international crossings and the spatial extent of associated movements (distance and areal extent) by pelagic fishes may be due in part to their differential use of coastal (on continental shelf, <200 m depth) and open ocean (off continental shelf, >200 m depth) ecosystems. Recent research suggests that displacement lengths (distance) and the type of movement (random versus directed) differs for large marine predators inhabiting coastal shelf versus open ocean ecosystems, with species occurring off the continental shelf showing more directed movements with larger displacement lengths^28^. Observed movement patterns for the eight species investigated largely support this premise. Species with the majority of daily positions in oceanic waters—which included blue marlin, white marlin, Atlantic bluefin tuna, and whale sharks—showed the greatest tendency to transit international borders with most crossings occurring in open ocean regions. In addition, these species traveled the farthest distances from initial tagging locations. In fact, nearly all of the long-term tag deployments (>150 days) for these four species showed bi-national connectivity, with 93–100% of the individuals crossing into the territorial waters of either Cuba or Mexico during their track. By contrast, the majority of daily positions for scalloped hammerheads and tiger sharks, and to a lesser degree yellowfin tuna, were inside or near the continental shelf break (~200 m isobath), and international crossings by these species were more limited. In fact, nearly all individuals from these three species showed residency to the U.S. GoM, which was not entirely unexpected because more limited displacement distances have been reported previously for each species^29–31^. The diverse range of resources and habitats on the continental shelf presumably promotes more complex, but less directed movements by these species^32^, often resulting in more restricted displacement from the initial tagging location. The close association of daily positions along the outer continental shelf for scalloped hammerheads and tiger sharks  is in accord with the coastal-open ocean movement hypothesis, and both species are commonly grouped into the “coastal shark management group” by fishery management organizations^33^.

For species showing strong bi- or tri-national population connectivity, the timing of international crossings and the periods of occurrence in the territorial waters of Cuba, Mexico, or the Caribbean Sea varied seasonally. Movements out of the U.S. GoM during summer and fall and into Mexico or areas off Cuba in the fall and winter were observed for several of the species investigated. Seasonal movement of pelagic fishes into the southern GoM and Caribbean Sea has been suggested previously for open ocean and coastal migratory species including blue marlin^16^, whale sharks^34,35^, king mackerel^36^ and tarpon^37^. Here, conspicuous fall/winter movements into the territorial waters of Mexico were observed for over half of the species investigated (blue marlin, white marlin, yellowfin tuna, whale sharks). In addition, a greater percentage of the daily positions for Atlantic bluefin tuna during the winter (i.e., beginning of ingress into the GoM spawning area) were in Mexico. However, by spring the majority of daily positions for Atlantic bluefin tuna were farther north in the U.S. GoM, which suggests that adults may move into the northern part of the basin (north of 26°N latitude) in the late spring as sea surface temperature (SST) starts to increase and the spawning period begins.

Seasonal changes in the distribution of marine fishes have been linked to external drivers, with individuals often attempting to reduce the amount of environmental variability experienced throughout the year^6^. Because the physicochemical environment at the same location in the GoM can vary markedly over the year, individuals may move to new locations to compensate or control for seasonal changes in external conditions. For pelagic fishes, shifts in their spatial and temporal distribution have been attributed to a variety of physicochemical factors including SST, salinity, chlorophyll *a*, and dissolved oxygen^17,26,38,39^, and such factors may be responsible for the convergence of seasonal movement patterns observed for blue marlin, white marlin, yellowfin tuna, and whale sharks. Daily positions of all four species were concentrated in the U.S. GoM during the spring and summer, with positions shifting farther south into the territorial waters of Mexico, Cuba, and the Caribbean Sea in the fall and winter. The following spring, individuals often moved back into the U.S. GoM, possibly indicating the closure of an annual migration loop between the northern and southern regions of this basin. One external factor that shows strong seasonal variability in the U.S. GoM is SST, and changes in this factor are known to influence the movement of pelagic fishes as well as other marine predators^1^. Given that mean SSTs in outer shelf and slope waters of the U.S. GoM can vary 6–8 °C between summer and winter seasons, it is possible that movement south into the territorial waters of Mexico by these species is an overwintering adaptation. In fact, occupying regions in the southern part of the basin during winter reduces the SST change experienced by up to 3–4 °C (NOAA Physical Oceanography Division, http://www.aoml.noaa.gov/phod/dhos/sst.php), possibly serving to moderate physiological stress for species exhibiting this type of seasonal movement pattern.

Convergence on the seasonal movement pattern described above may also be attributed to intrinsic incentives related to reproduction and/or energetics^6^. A quintessential example of a species displaying directed, long-distance movements to support reproductive activities is the Atlantic bluefin tuna^40,41^. This temperate tuna commonly resides in the North Atlantic Ocean but migrates to the tropical waters of the GoM to spawn^17,42^. Entry into the warmer and highly productive waters of the GoM presumably supports rapid growth of larvae and early juveniles (optimal SST ~24–28 °C)^25^, which may lead to higher survival and recruitment potential for the early life stages even though the upper range of SSTs encountered in the spring and summer are near the cardiac capacity of spawning adults^43^. Bottom up processes that enhance the growth and survival of Atlantic bluefin tuna larvae and other pelagic species is also a likely explanation for the presence of higher order consumers such as whale sharks, billfishes, and tunas. These species presumably move into this region to take advantage of abundant prey resources linked to nutrient loading and associated production from the Mississippi River. In fact, the presence of whale sharks in the U.S. GoM during the spring and summer is potentially related to energetics with this species gaining a trophic advantage by feeding on eggs and larvae (top down control by predator) produced by both invertebrates and fishes in these highly productive waters. Interestingly, movements into territorial waters farther south off Cuba, Mexico, and into the Caribbean Sea may also be driven by favorable foraging conditions and associated energetic benefits because whale sharks commonly aggregate and show site fidelity to known mass spawning areas^44–46^. Our findings demonstrate that several species converge on a common seasonal movement pattern between summer (U.S. GoM) and winter (Mexico) locations within the basin, and intrinsic motivations for such movements appear to be associated with both bottom up and top down processes.

While the nature and timing of spatial shifts in distribution is essential information for management, areas of international exchange are typically poorly defined, and crossing locations defined here highlight priority areas for billfish, tuna, and shark conservation. Similarities were observed between our two multispecies groups, with important areas of exchange located in the central GoM across large expanses of the open ocean. One area in particular was identified between the two high seas zones in the GoM as an important crossing location for both billfishes/tunas and sharks. Although a variety of extrinsic factors are known to influence ocean navigation and migration pathways of vertebrate fauna (e.g., currents, fronts, magnetic fields^47^), this multispecies crossing location occurs in oceanic waters that are relatively homogenous and less complex in physicochemical conditions than areas on or near the continental shelf. As a result, less directed or more free-ranging movements by billfishes, tunas, and sharks may be responsible for elevated levels of border crossings observed in this area. Although oceanic waters extend east of this border crossing location, transnational movements between Mexico and the U.S. east of 88°W longitude were less evident (Fig. 6). The western margin of the northward flowing Loop Current in the GoM often resides near 88°W longitude or close to the easternmost high seas region of the basin. Because billfishes, tunas, and pelagic sharks often use frontal boundaries or areas near the outer margins of major currents^48,49^, movement along the edge of this mesoscale feature may explain the high concentration of international crossings observed in this general area. Farther east, individuals would be within this mesoscale feature and traveling against the prevailing current and in less productive waters, which may explain the more limited degree of north to south movement between the U.S. and Mexico at 87–85°W longitude. We also observed a second hotspot of exchange for both taxonomic groups in the western GoM, with the primary crossing area being on the continental shelf for sharks and in deeper waters for the billfish and tuna group. This finding further accentuates the on-shelf affinity by several of the sharks investigated, while blue marlin, white marlin, Atlantic bluefin tuna, and yellowfin tuna were more commonly observed on the outer continental shelf or in oceanic waters off the shelf.

One caveat to characterizing movements of pelagic fishes using data from multiple tagging efforts and different tagging platforms is that distance traveled and the number of areas/regions visited (i.e., international crossings) is often a function of both the release location and tag configuration (e.g., deployment duration). As expected, shorter tracking periods are often associated with more limited displacement distances^16,50^. Moreover, daily positions are often concentrated around release locations, particularly for species displaying more limited movements as shown for certain shark species (Fig. 4). It is also important to note that individuals tracked for shorter periods of time may not be representative of an individual’s movement capacity because post-release behavioral modifications related to capture and handling stress may extend up to 60 days^51^. Recognizing these potential sources of bias is fundamental to developing accurate representations for metadata derived from independent tagging efforts. Fortunately, our findings on population connectivity and international crossings were consistent between pooled metadata for each species and the more limited dataset comprised only of long-term tags (>150 days at liberty) (Table 2), supporting the assertion that our characterization of population connectivity using pooled data from multiple tagging campaigns was appropriate for the pelagic predators assessed. Nevertheless, we acknowledge that different tagging platforms and modeling frameworks used to track these pelagic fishes may have affected the precision, accuracy, and number of daily location estimates^52^. Moreover, future studies with extended tracking periods and larger samples sizes are critically needed to develop a more holistic understanding of international exchanges, which will guide future management and rebuilding efforts for these ecologically and economically valuable species.

The scale of management (unit stock) is inherently linked to animal movement, and changes in the spatial structure can alter population and community dynamics^53^. Even low rates of movement across international boundaries or between management zones can compromise our ability to effectively assess population status and achieve sustainable management^54,55^. The current study emphasizes the range of possibilities regarding the spatial distribution and movement of large pelagic fishes common to the GoM, and plainly shows a need for cooperative fisheries management among Cuba, Mexico, and the U.S. for many of the highly migratory species that inhabit these waters. Research to improve our understanding of the drivers (intrinsic and extrinsic) of both temporary movements and longer-term, directed seasonal migrations across international boundaries will ultimately lead to more informed characterizations of the spatial structure of billfish, tuna, and shark populations within the GoM. Since the majority of the species assessed in this study are IUCN red listed as endangered or vulnerable, failure to adequately define the spatial dynamics of GoM populations and the mechanisms underlying their movement will further compromise the ability of resource managers to quantify the impacts of both anthropogenic and natural disturbances that are common to this region (e.g. oil spills, hurricanes), as well as forecast the impacts of a changing environment (climate change) on these species.

## Methods

Similar to other multispecies assessments that highlight the movement pathways of apex predators^1^, this study was based on pooled data from several electronic tagging efforts and platforms (Table 1). Daily position estimates were generated using smart position tags (SPOTs), pop up archival tags (PATs), and archival implants tags (AITs). Details regarding tag programming and attachment varied among species but followed previously described protocols^15–17,56,57^. Because the aim of this study was to characterize spatial and temporal shifts in the distribution of pelagic predators residing in U.S. GoM, we relied exclusively on tagging conducted in this region for seven of eight species investigated. For Atlantic bluefin tuna, a fraction of the daily positions was again derived from tagging performed in the U.S. GoM; however, natal homing is well developed for this species and individuals return to the GoM to spawn^42^. Their directed movements into this basin also allowed for the incorporation of additional tagging conducted outside the U.S. GoM (Canada) to explore transnational movements and the significance of international crossings by individuals after moving into the basin. All tagging was performed in accordance with relevant guidelines and regulations of institutional animal care and use committees (IACUC) and tagging protocols were approved by Texas A&M University (TAMU) and Texas A&M University-Corpus Christi (TAMUCC). IACUC approved animal use protocols relevant to this study included TAMU 2007-168, TAMU 2013-0221, TAMU 2017-0056, and TAMUCC 08-15.

The accuracy and precision of daily position estimates (latitude and longitude) from different electronic tagging platforms varies, with only SPOTs transmitting directly to the Argos satellite system during the tracking period for near real-time positions. Both PATs and AITs include light and temperature sensors for deriving light-based geolocation. The accuracy of daily positions based on ARGOS satellite transmissions from SPOTs (or PATs after pop up to the surface) are often highly accurate (<500 m^58^); however, light-based positions derived from PATs and AITs are known to have markedly larger estimates of uncertainty, especially for estimating latitude around equinox phases^59^. Daily positions from SPOTs used in this study were filtered to remove poor location class (Z) positions, with highest quality position estimates used each day for all individuals outfitted with these tags. For PATs and AITs, a variety of approaches are commonly used to estimate daily position using light-based models. Wildlife Computers PATs (e.g. MK10, Mini-PAT) fit a subset of light levels at sunrise and sunset to a sun elevation model, while Microwave Telemetry PATs (e.g. X-Tag) rely on a proprietary algorithm to extract the timing of sunrise and sunset^60^. Light-based location from Lotek AITs (LAT 2000 series) is based on template fitting to irradiance using a geophysical model of twilight^61^. Also, data included in our assessment utilized state-space models that implement the Kalman filter (KFTRACK and UKFSST packages; http://positioning.github.io) to reduce uncertainty and further refine daily positions^62^. Although the accuracy and resolution of the initial or post-processed daily positions may differ among the approaches used, all are assumed to yield suitable locations for examining movement at the scale being investigated here.

Given that our intent was to describe the range of migratory behaviors displayed by billfishes, tunas, and pelagic sharks in the U.S. GoM, data from multiple tagging periods (years) were combined to characterize movements and spatial distributions of each species. Even though migration patterns of these and other highly migratory species are known to vary from year to year^26,35,63^, interannual variability was not assessed because most tagging efforts in this region did not tag sufficient numbers of individuals across multiple years to allow for such comparisons^64–66^. Moreover, certain intrinsic factors (e.g., sex, age) are known to influence the migration patterns of marine teleosts^16,27^ and sharks^67^, but evaluating these factors was beyond the scope of this paper.

Metadata similar to our multispecies dataset are useful for characterizing transnational movements of marine megafauna, often between foraging and spawning areas^24,28^, and the integration of data derived from different tagging platforms have also proven useful for elucidating seasonal shifts displayed by pelagic predators^1^. In the current paper, daily positions of each species were partitioned into four quarterly seasonal periods: January–March (winter), April–June (spring), July–September (summer), and October–December (fall) to explore intra-annual patterns of movement for each of the selected species. The seasonal use of territorial waters off Cuba, Mexico, U.S. GoM, and adjacent areas was assessed by calculating the percent occurrence of individuals in 6 designated regions: (1) U.S. GoM (includes Florida Keys), (2) Mexico (all territorial waters in the GoM plus waters from the Yucatan Channel to Xcalak), (3) high seas areas in the GoM, 4) Cuba (all territorial waters), (5) U.S. waters in the Atlantic Ocean outside GoM, and (6) all remaining areas of the Atlantic Ocean (i.e., locations outside regions 1–5 including the Caribbean Sea) (Fig. 1).

Daily positions were presented along with information on the primary tagging areas for each species (based on 95% kernel utilization distributions [KUD] from deployment locations) within ArcGIS 10.2.2. The spatial configuration and extent of the 95% KUDs for tagging areas varied among species, and KUDs were included to acknowledge inherent bias in the spatial distribution of daily positions due to the geographic location of tagging activities (Figs 2 and 4). Territorial boundaries (established or virtual) of Cuba, Mexico, and the U.S. were accessed from https://maritimeboundaries.noaa.gov and shape files were imported into ArcGIS for assigning each daily position to a region (1–6). Percent occurrence to each region was determined by dividing the number of daily positions in a region to the total number of daily positions for each species. Multispecies crossing areas were also described for two taxonomic groups (billfishes/tunas and sharks) in an effort to describe the geographic location(s) where the majority of transnational movements occurred. These areas of exchange or pathways to other countries were based on daily positions 10 days before and 10 days after each international crossing, and determined using a biased random bridge approach^68^ to derive movement-based kernel density estimates (MKDE) based on daily position estimates. We then summed MKDEs of individuals to visualize multispecies crossing areas for each group. The frequency of open ocean (>200 m depth) versus coastal (<200 m depth) crossings between the two multispecies groups was examined with a chi-square test.

The relationship between days at liberty and international crossings was also investigated for one representative teleost (blue marlin) and one shark (whale shark). We selected these two species because both displayed non-resident behavior and regularly moved to areas outside the U.S. GoM. Also, these two species had the greatest number of daily positions for individuals tagged in the U.S. GoM. Days at liberty were binned into 10-d periods for each individual and then the percent of individuals displaying international movement was estimated for each 10-d bin up to 200 days. Once an individual crossed into the territorial waters of either Cuba or Mexico, it retained the international migrant designation for the remainder of the deployment period. Locally weighted scatterplot smoothing (LOWESS) was used as the non-parametric regression method to describe non-linear relationships between days at liberty and the likelihood of an international crossing for both species.

## Supplementary information

Dataset 1

## Data Availability

Daily position estimates for each species are available in Supplementary Data File S1. Additional details regarding dataset used in the current study are available from the corresponding author upon request.
